# Elevated FGF23 Levels in Mice Lacking the Thiazide-Sensitive NaCl cotransporter (NCC)

**DOI:** 10.1038/s41598-018-22041-1

**Published:** 2018-02-26

**Authors:** Ganesh Pathare, Manuel Anderegg, Giuseppe Albano, Florian Lang, Daniel G. Fuster

**Affiliations:** 1 Division of Nephrology and Hypertension, Bern University Hospital, University of Bern, Bern, Switzerland; 2 Institute of Biochemistry and Molecular Medicine, University of Bern, Bern, Switzerland; 3 Swiss National Centre of Competence in Research (NCCR) Transcure, University of Bern, Bern, Switzerland; 4 Department of Cardiology, Vascular Medicine and Physiology, Eberhard-Karls-University of Tübingen, Tübingen, Germany; 5 Department of Molecular Medicine II, Medical Faculty, Heinrich Heine University, Düsseldorf, Germany

## Abstract

Fibroblast growth factor 23 (FGF23) participates in the orchestration of mineral metabolism by inducing phosphaturia and decreasing the production of 1,25(OH)_2_D_3_. It is known that FGF23 release is stimulated by aldosterone and extracellular volume depletion. To characterize this effect further in a model of mild hypovolemia, we studied mice lacking the thiazide sensitive NaCl cotransporter (NCC). Our data indicate that NCC knockout mice (KO) have significantly higher FGF23, PTH and aldosterone concentrations than corresponding wild type (WT) mice. However, 1,25(OH)_2_D_3_, fractional phosphate excretion and renal brush border expression of the sodium/phosphate co-transporter 2a were not different between the two genotypes. In addition, renal expression of FGF23 receptor FGFR1 and the co-receptor Klotho were unaltered in NCC KO mice. FGF23 transcript was increased in the bone of NCC KO mice compared to WT mice, but treatment of primary murine osteoblasts with the NCC inhibitor hydrochlorothiazide did not elicit an increase of FGF23 transcription. In contrast, the mineralocorticoid receptor blocker eplerenone reversed excess FGF23 levels in KO mice but not in WT mice, indicating that FGF23 upregulation in NCC KO mice is primarily aldosterone-mediated. Together, our data reveal that lack of renal NCC causes an aldosterone-mediated upregulation of circulating FGF23.

## Introduction

Fibroblast growth factor‐23 (FGF23) is a bone derived phosphate and 1,25-dihydroxyvitamin D_3_ (1,25(OH)_2_D_3_) regulating hormone which is secreted by osteoblasts and osteocytes^1^. Fibroblast growth factor receptor 1 (FGFR1) is the principal mediator of the effects of FGF23^2,3^. Furthermore, α-Klotho, an antiaging protein, binds to FGFR1 and increases the affinity of FGFR1 for FGF23^4^. In the kidney, circulating FGF23 reduces phosphate reabsorption from primary urine through a direct downregulation of the sodium phosphate co‐transporter 2 a (NaPi2a) in renal proximal tubular epithelial cells^5^. In addition, FGF23 inhibits the formation of 1,25(OH)_2_D_3_ by down-regulating renal 1α hydroxylase (Cyp27b1) and stimulating 1,25(OH)_2_D_3_ inactivation by upregulation of 25-hydroxyvitamin D 24-hydroxylase (Cyp24a1)^6^. Increased FGF23 levels may be pathophysiologically relevant^7,8^, as they are correlated with accelerated disease progression and increased morbidity and mortality in several clinical disorders including cardiac failure^9,10^, acute renal failure^11^, chronic kidney disease^4,10,12^, diabetic nephropathy^13^ and hepatic failure^14^. FGF23 levels rise during chronic kidney disease (CKD) progression and highest levels are found in patients with CKD stage V. Our recent work indicates that FGF23 levels begin to rise much earlier during CKD progression than previously appreciated, even before the establishment of clinically evident CKD^15^.

Thiazide diuretics act by blocking the thiazide-sensitive NaCl cotransporter (NCC), apically localized in epithelial cells lining the distal convoluted tubule (DCT)^7^. Recent data suggest that FGF23 directly activates NCC in the DCT, thereby inducing volume expansion, hypertension, and subsequently left ventricular hypertrophy^16^. Interestingly, the thiazide diuretic hydrochlorothiazide prevented these presumed off target effects. Conversely, changes in volume status may modulate FGF23 concentrations, which is evident at least in patients undergoing hemodialysis^17^. This suggests the presence of a feedback loop, where FGF23 increases volume load, and an increase in volume may reduce FGF23. Therefore, hypovolemia may in turn increase FGF23.

Loss-of- function mutations in NCC cause Gitelman’s syndrome (OMIM 263800), a condition characterized by sodium wasting and hypotension^8,9^. Extracellular volume is decreased and aldosterone levels are increased by either pharmacological inhibition or genetic deletion of NCC^8,9,18,19^. Aldosterone upregulates FGF23 levels in osteoblasts^20^. Aldosterone is in part effective by augmenting store operated Ca^2+^ entry (SOCE) with subsequent increase of cytosolic Ca^2+ ^^21^, a powerful stimulator of FGF23 transcription^22^. The aldosterone-mediated FGF23 release presumably contributes to the up-regulation of FGF23 plasma concentration by salt depletion^20^, lithium treatment^23^, non-ischemic cardiac disease^10^ and early chronic kidney disease^10^. With these considerations in mind, we hypothesized that NCC activity modulates circulating FGF23 levels indirectly via extracellular volume and aldosterone secretion.

## Results

### NCC KO mice exhibit higher circulating FGF23 levels and higher FGF23 transcript in bone

**Figure 1:**
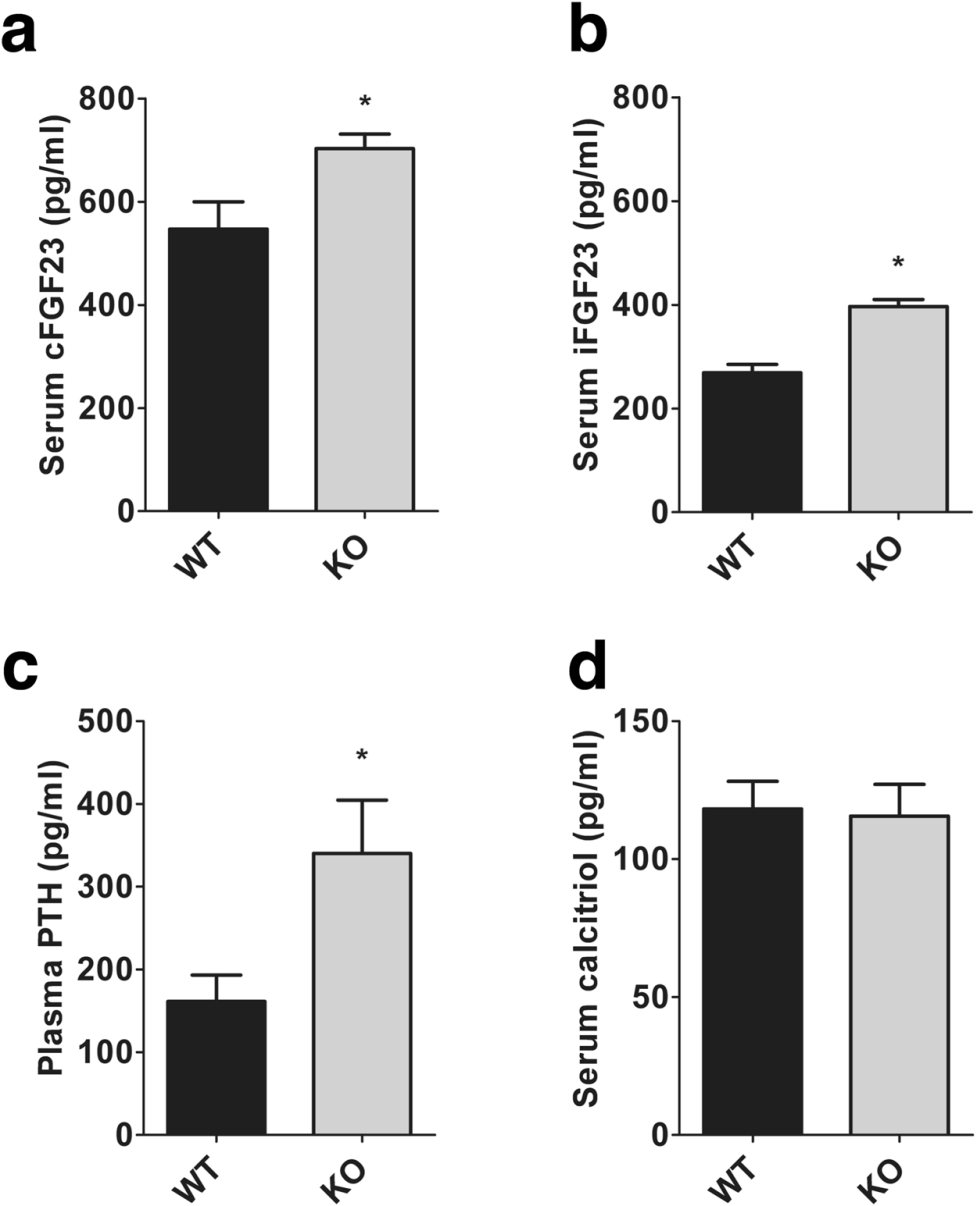
Blood concentrations of cFGF23, iFGF23, PTH and 1,25(OH)_2_D_3_ in WT and NCC KO mice. Arithmetic means ± SEM (n = 9–12) of serum cFGF23 (**a**), serum iFGF23 (**b**), plasma PTH (**c**) and serum 1,25(OH)_2_D_3_ (**d**) in WT (grey bars) and NCC KO (black bars) under normal diet. *(p < 0.05) indicates significant difference from NCC WT mice.

We first measured mineral metabolism markers in WT and NCC KO mice. To this end, both C-terminal FGF23 (cFGF23) and intact FGF23 (iFGF23) levels were determined by ELISA. As shown in Fig. 1, the cFGF23 and iFGF23 levels were significantly higher in NCC KO mice than in WT mice on standard rodent chow. Both cFGF23 and iFGF23 were elevated to a similar degree. We also observed a significant increase of PTH in NCC KO mice compared to WT mice. 1,25(OH)_2_D_3_ levels, however, were similar in both groups of mice.

**Figure 2:**
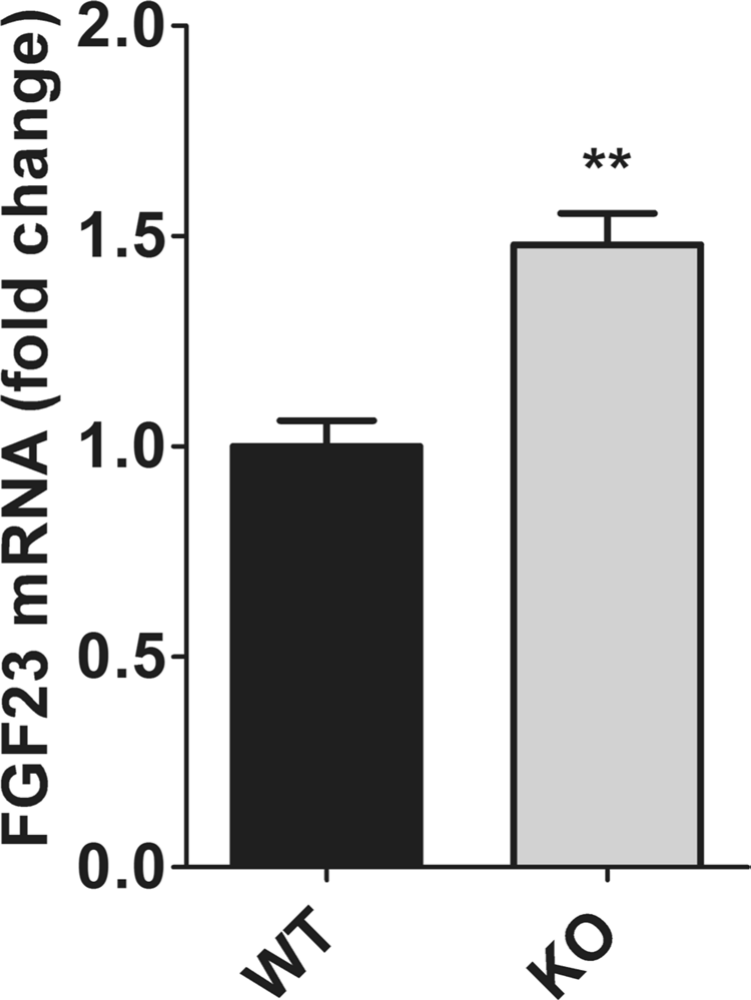
FGF23 mRNA levels in femora of WT and NCC KO mice. Arithmetic means ± SEM (n = 6–8) of FGF23 mRNA levels in femora obtained from WT (black bars) and NCC KO mice (grey bars) under normal diet. **(p < 0.01) indicates significant difference from NCC WT mice.

Bone is the primary site of FGF23 formation. Therefore, FGF23 transcript levels were quantified in femora of WT and NCC KO mice. As shown in Fig. 2, FGF23 mRNA was significantly higher in NCC KO mice than littermate WT mice.

### Phosphate homeostasis is unaltered in NCC KO mice

**Figure 3:**
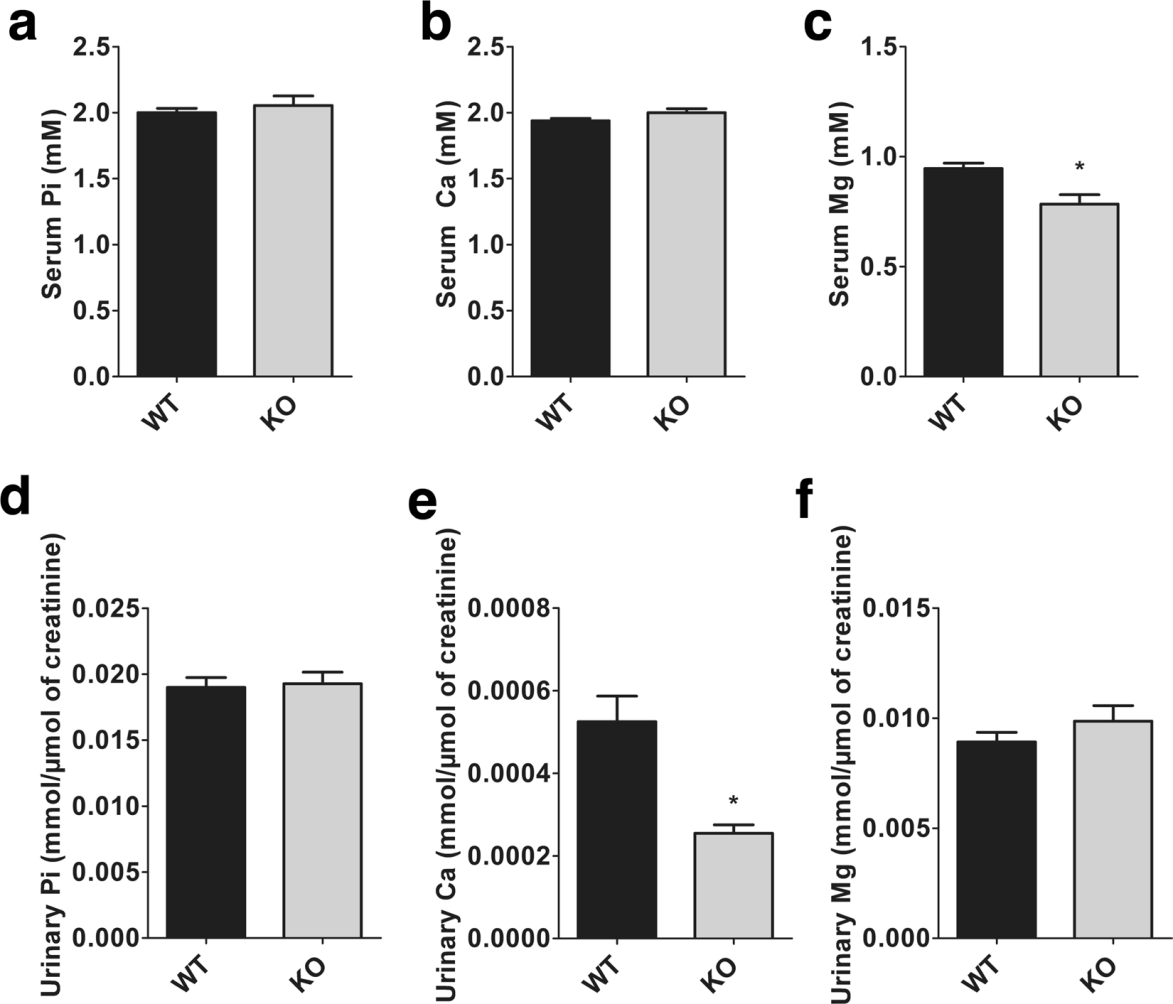
Blood and urinary phosphate (Pi), calcium (Ca) and magnesium (Mg) in WT and NCC KO mice. (**a**–**c**) Arithmetic means ± SEM (n = 9–12) of serum Pi (**a**), Ca (**b**) and Mg (**c**) in WT (black bars) and NCC KO mice (grey bars) under normal diet. (**d**–**f**) Arithmetic means ± SEM (n = 9–12) of urinary Pi (**d**), Ca (**e**) and Mg (**f**) normalized with creatinine in WT (black bars) and NCC KO mice (grey bars) under normal diet. *(p < 0.05) indicates significant difference from WT mice.

We next sought to determine the impact of elevated FGF23 levels on calcium and phosphate homeostasis. As illustrated in Fig. 3, serum phosphate and calcium concentrations were not significantly different between NCC KO mice and WT mice. As previously reported^24^, the serum magnesium concentration was found to be significantly reduced in NCC KO mice. Absolute urinary phosphate and magnesium excretion were similar in NCC KO and WT mice, whereas the urinary calcium excretion was significantly lower in NCC KO mice. Fractional excretion (FE) of calcium was significantly lower (WT: 0.56 ± 0.08%; KO: 0.30 ± 0.05%; p = 0.010) and FE of magnesium was significantly higher in NCC KO mice (WT: 20.13 ± 1.67%; KO: 28.14 ± 3.09%; p = 0.029). No significant difference was observed in FE of phosphate between the two genotypes of mice (WT: 19.72 ± 1.80%; KO: 21.39 ± 2.13%; p = 0.553).

**Figure 4:**
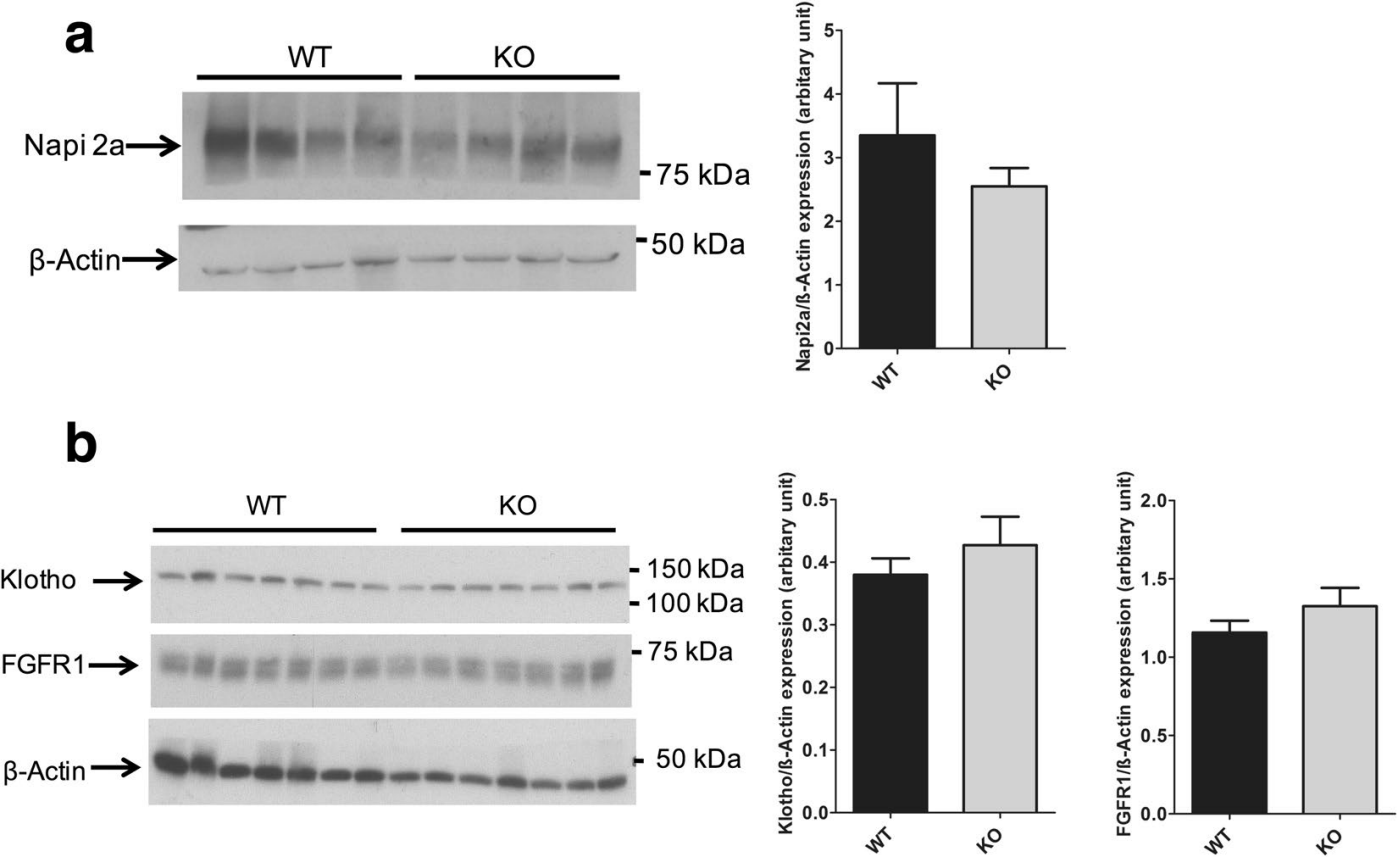
Brush border abundance of Napi2a and renal expression of α-Klotho and FGFR1. (**a**) Original western blot of Napi2a and beta actin in BBM isolated from NCC WT and KO kidneys. Quantification of Napi2a expression in WT and KO kidneys. (**b**) Original western blot of α-Klotho, FGFR1 and beta actin in total kidney lysate from NCC WT and KO kidneys. Quantification of α-Klotho and FGFR1 expression in WT and KO kidneys.

To assess if higher FGF23 levels affect the apical abundance of proximal tubular NaPi2a, we isolated brush border membrane vesicles (BBMV) from NCC WT and KO mice and immunoblotted for NaPi2a. As shown in Fig. 4a, NaPi2a expression in BBMVs isolated from NCC KO mice and WT mice were similar. We next also assessed expression of FGFR1 and its co-receptor α-Klotho in NCC WT and KO total kidney lysates. As illustrated in Fig. 4b, expression of α-Klotho and FGFR1 were similar in kidneys of both groups of mice.

### Inhibition of NCC does not cause an increase FGF23 transcript in primary osteoblasts

**Figure 5:**
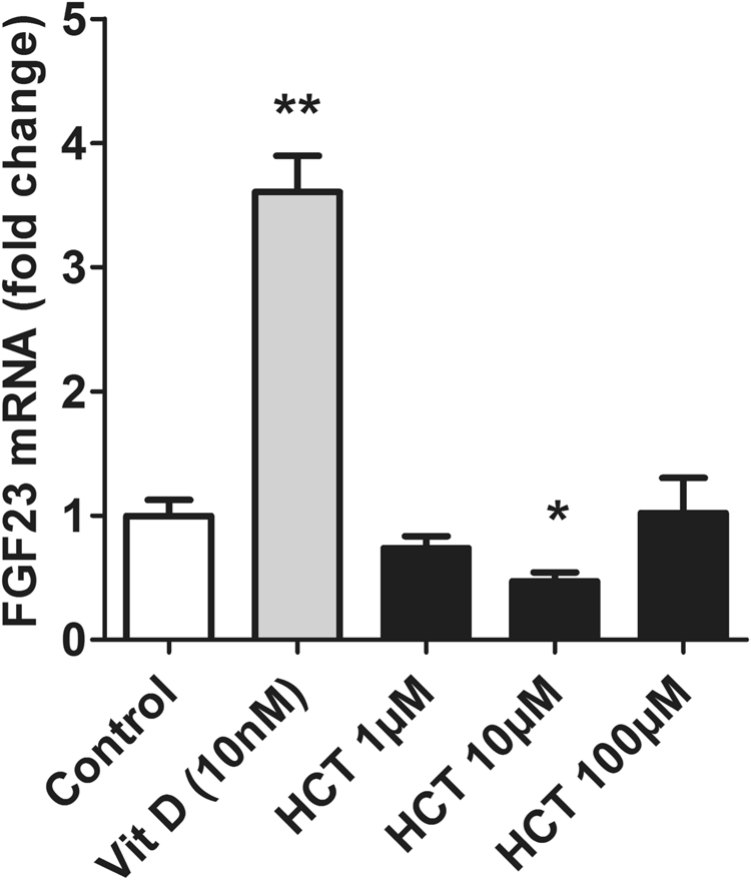
Effect of hydrochlorothiazide (HCT) treatment on FGF23 expression in murine primary osteoblast. Arithmetic means ± SEM (n = 5–6) of FGF23 mRNA levels in murine primary osteoblasts following treatment with vehicle (ethanol; control), 10 nM 1,25(OH)_2_D_3_ or 1, 10, 100 μM HCT. *(p < 0.05) for ctrl vs 1,25(OH)_2_D_3_, HCT 1, 10, 100 μM. FGF23 transcript levels are expressed relative to the level of the GAPDH control gene.

NCC is expressed in osteoblasts^25,26^ and therefore may contribute to the regulation of FGF23 release. To explore this possibility, we tested whether the NCC inhibitor hydrochlorothiazide was able to up-regulate FGF23 transcript levels in primary murine osteoblasts. We isolated primary osteoblasts from neonatal murine calvaria. Primary osteoblasts were treated with vehicle (negative control), 10 nM 1,25(OH)_2_D_3_, (positive control) or three different concentrations of the thiazide diuretic hydrochlorothiazide (1, 10 or 100 µM) for 24 hrs. As illustrated in Fig. 5, FGF23 transcript was significantly upregulated by 1,25(OH)_2_D_3_ treatment. In contrast, FGF23 mRNA remained unchanged following treatment with 1 µM or 100 µM hydrochlorothiazide, whereas 10 µM hydrochlorothiazide caused a small decrease. Hence, increased circulating FGF23 levels in NCC KO mice seem not to be due to NCC deficiency in FGF23 producing osteoblasts.

### Mineralocorticoid receptor blockade reduces FGF23 levels in NCC KO but not WT mice

**Figure 6:**
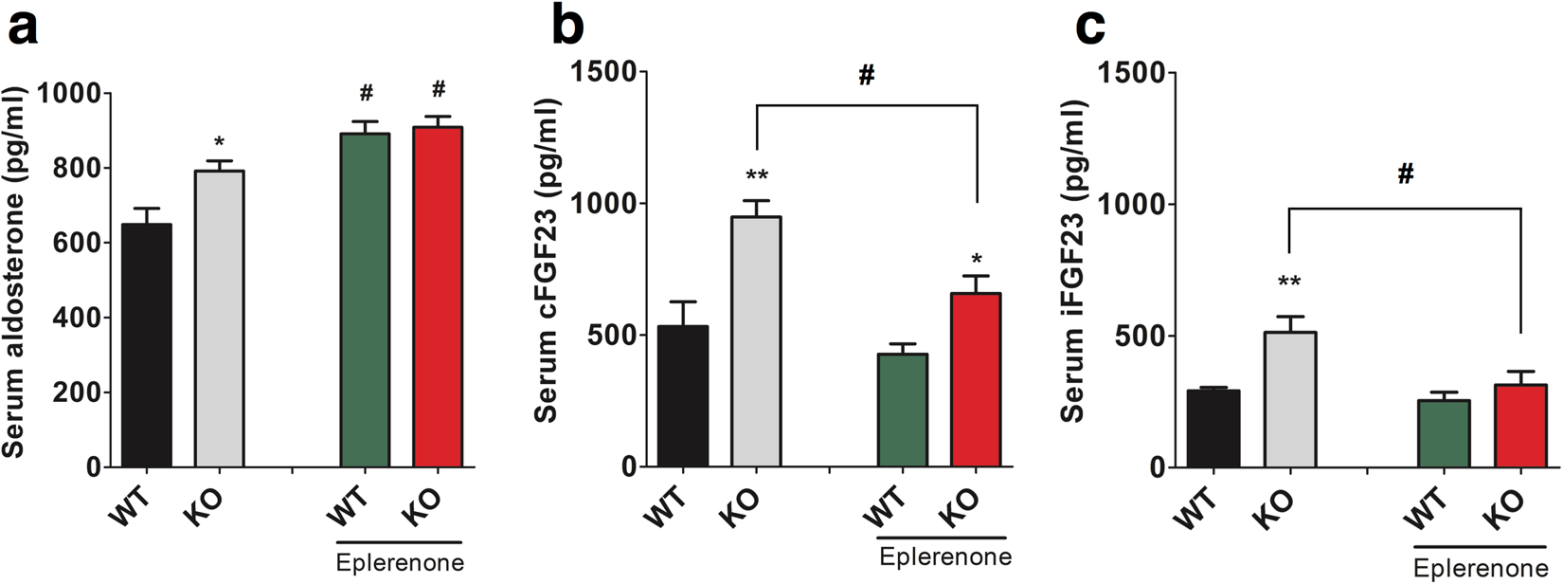
Effect of eplerenone on circulating aldosterone, cFGF23 and iFGF23 levels. (**a**–**c**) Arithmetic means ± SEM (n = 6–7) of serum aldosterone (**a**), serum cFGF23 (**b**), and serum iFGF23 (**c**) in NCC WT mice NCC KO under baseline and following oral eplerenone treatment for 7 days (right bars). *(p < 0.05) indicates significant difference from NCC WT mice, ^#^(p < 0.05) indicates significant difference from eplerenone diet.

Under regular chow diet, aldosterone levels are higher in NCC KO mice (Fig. 6a). In order to test if aldosterone is mediating the elevation of FGF23 levels in NCC KO mice, we treated NCC WT and KO mice with the specific mineralocorticoid receptor blocker eplerenone for 7 days. Blood samples were taken prior and after the treatment. Serum aldosterone, cFGF23 and iFGF23 were measured. As expected, aldosterone levels were significantly increased in both genotypes after eplerenone treatment (Fig. 6a). Eplerenone treatment significantly reduced cFGF23 and iFGF23 in NCC KO but did not affect FGF23 levels in WT mice (Fig. 6b,c). The difference in iFGF23 between WT and NCC KO mice was completely abolished by eplerenone treatment whereas cFGF23 levels remained higher in NCC KO mice.

### Effect of phosphate deficient diet on mineral metabolism in NCC KO mice

**Figure 7:**
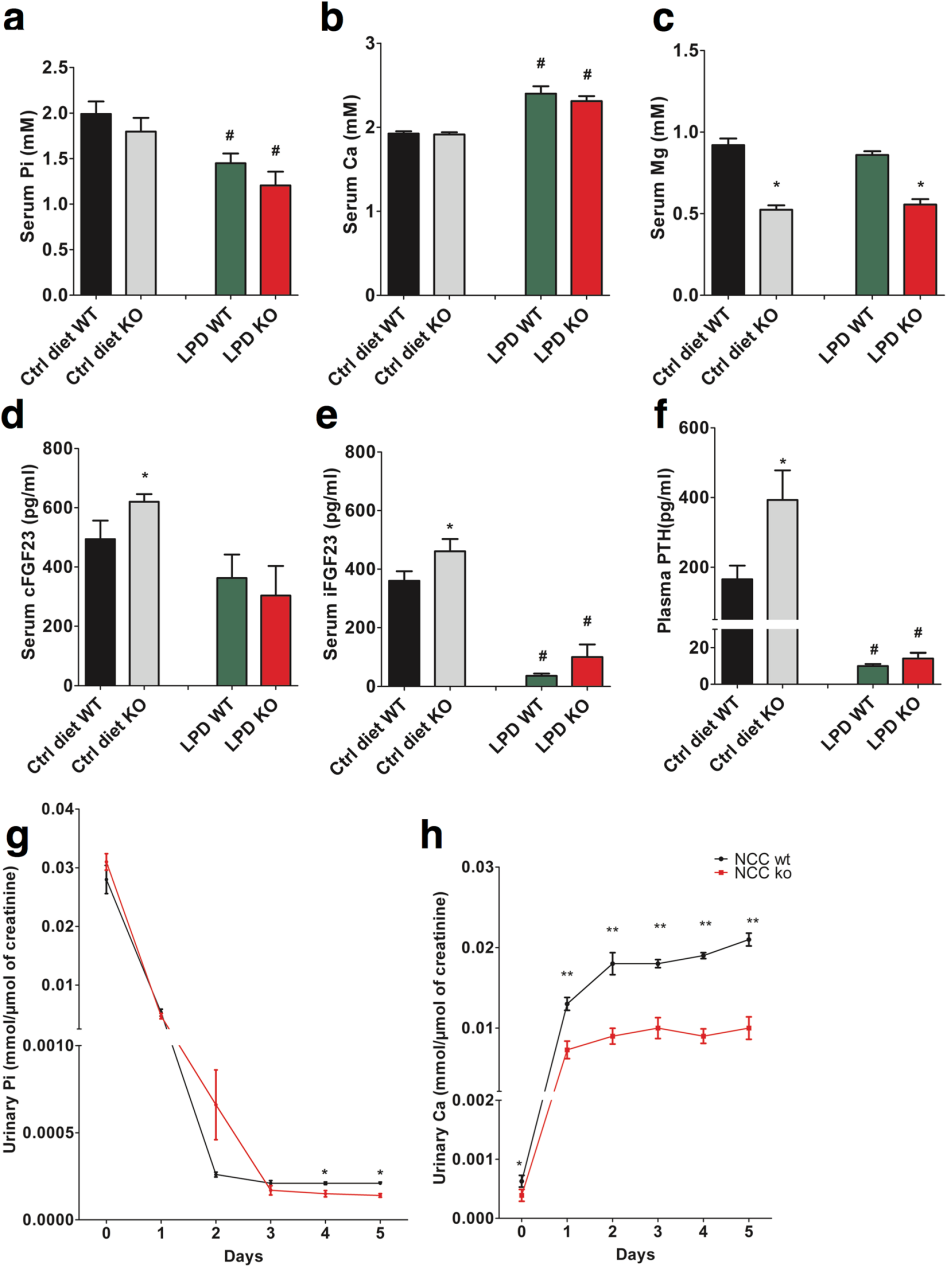
Effect of dietary phosphate depletion on blood and urinary electrolytes and circulating cFGF23, iFGF23 and PTH levels in WT and NCC KO mice. (**a**–**f**) Arithmetic means ± SEM (n = 6–7) of serum phosphate (**a**), Ca (**b**) and Mg (**c**) levels. Serum cFGF23 (**d**), serum iFGF23 (**e**), and plasma PTH (**f**) in NCC WT mice (grey bars) and NCC KO (black bars) under normal diet (Ctrl diet, left bars) and following dietary phosphate depletion for 5 days (LPD, right bars). *(p < 0.05) indicates significant difference from NCC WT mice, ^#^(p < 0.05) indicates significant difference from normal diet. (**g,h**) Arithmetic means ± SEM (n = 6–7) of urinary phosphate excretion (**g**) and urinary calcium excretion (**h**) in NCC KO and wild type mice under normal diet (Day 0) and following dietary phosphate depletion (Day 1–5). *(p < 0.05), **(p < 0.01) indicate significant difference from NCC WT mice.

We next subjected WT and NCC KO mice to a phosphate deficient diet for a total of 5 days to create a negative phosphate balance and stress the phenotype of NCC KO mice. As shown in Fig. 7a–c, the phosphate deficient diet elicited in both genotypes a significant decrease of the serum phosphate concentration and a significant increase of the serum calcium concentration. Serum magnesium was not significantly modified by a low phosphate diet. After a low phosphate diet, the difference in cFGF23 levels between WT and NCC KO mice disappeared, the absolute decrease of cFGF23 in both groups of mice, however, was not significant (Fig. 7d). On the other hand, a low phosphate diet significantly decreased iFGF23 and PTH concentrations in both genotypes and dissipated the differences of iFGF23 and PTH between WT and KO mice (Fig. 7e,f). In both groups of mice, a low phosphate diet was followed by a sharp decline of urinary phosphate excretion and a marked increase of urinary calcium excretion (Fig. 7g,h). Urinary phosphate excretion was significantly lower in NCC KO mice on day 4 and 5 compared to NCC WT mice (Fig. 7g). Urinary calcium excretion remained significantly lower in NCC KO mice than in WT mice throughout the experiment. However, the difference between two genotypes was significantly amplified by a low phosphate diet (Fig. 7h).

### Effect of phosphate deficient diet on serum aldosterone and electrolytes homeostasis

**Figure 8:**
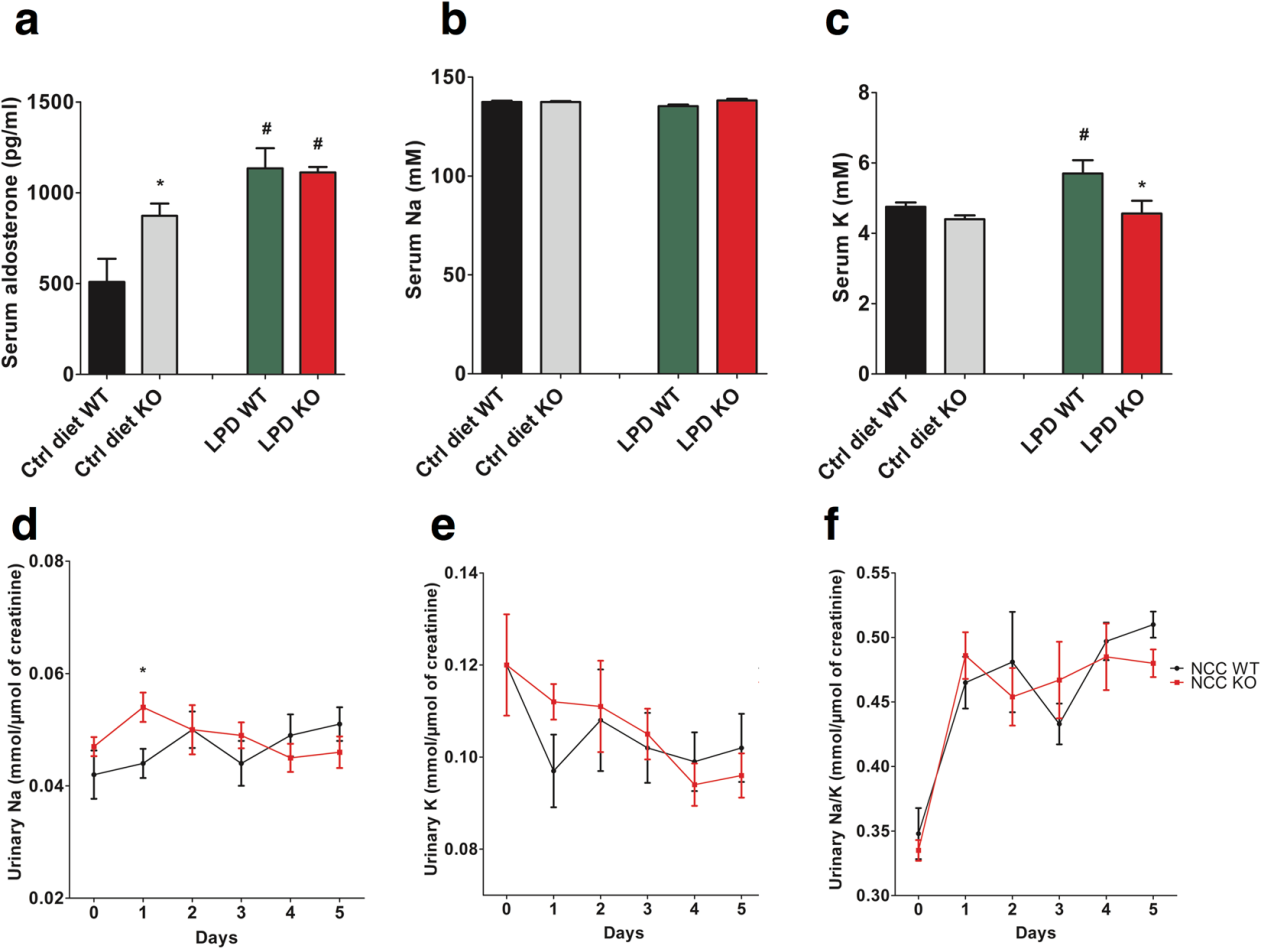
Effect of dietary phosphate depletion on blood and urinary electrolytes and circulating aldosterone levels in WT and NCC KO mice: (**a**–**c**) Arithmetic means ± SEM (n = 6–7) of serum aldosterone (**a**), Na (**b**) and K (**c**) levels in NCC WT mice (black bars) and NCC KO (grey bars) under normal diet (Ctrl diet, left bars) and following dietary phosphate depletion for 5 days (LPD, right bars). *(p < 0.05) indicates significant difference from NCC WT mice, ^#^(p < 0.05) indicates significant difference from normal diet. (**d**–**f**) Arithmetic means ± SEM (n = 6–7) of urinary Na excretion (**d**) urinary K excretion (**e**) and the ratio of urinary Na to K excretion (**f**) in NCC KO and WT mice under normal diet (Day 0) and following dietary phosphate depletion (Day 1–5). *(p < 0.05) indicate significant difference from NCC WT mice.

To explore if a low phosphate diet influences electrolyte homeostasis, serum and urinary sodium and potassium as well as aldosterone serum levels were determined at baseline and during the low phosphate diet. As shown in Fig. 8a, under a control diet, serum aldosterone was significantly higher in NCC KO mice compared to WT mice, as expected. Phosphate depletion induced an increase of serum aldosterone in both genotypes and dissipated the differences between the genotypes. Serum sodium and was not significantly different between the genotypes and was not significantly modified by a phosphate deficient diet (Fig. 8b). Urinary sodium excretion was significantly higher in NCC KO than WT mice at day one after initiation of a phosphate depleted diet (Fig. 8d). Phosphate depletion induced a significant increase of serum potassium in WT but not in NCC KO mice (Fig. 8c). Under low phosphate diet but not regular chow, NCC KO mice displayed reduced serum potassium compared to WT mice. The phosphate-deficient diet tended to decrease urinary potassium output in both genotypes, an effect, however, not reaching statistical significance (Fig. 8e). The urinary sodium to potassium ratio was similar in both groups of mice throughout the experiment (Fig. 8f).

## Discussion

The present observations reveal that deficiency of distal tubular NCC elicits an increase of circulating FGF23. Increased FGF23 levels in NCC KO mice are not accompanied by a reduction of circulating 1,25(OH)_2_D_3_, renal phosphate reabsorption or brush border expression of the sodium/phosphate co-transporter 2 a, suggesting renal resistance to the action of FGF23. Experiments with the mineralocorticoid receptor blocker eplerenone furthermore suggest that the observed increase in FGF23 levels in NCC KO mice is primarily a result of the volume depletion-induced secondary hyperaldosteronism.

Aldosterone works by up-regulating store operated Ca^2+^ entry (SOCE) with subsequent increase of cytosolic Ca^2+^ concentration^20,21^, a powerful stimulator of FGF23 transcription^22^. Renal tubular NaCl wasting and subsequent stimulation of aldosterone release possibly account further for the enhanced FGF23 levels in gene targeted mice expressing WNK-resistant SPAK^27,28^ or OSR1^29,30^. FGF23 serum levels are increased in heart failure^9,10^, acute renal failure^11^, chronic kidney disease^4,10,12^, diabetic nephropathy^13^ and hepatic failure^14^. In each of these disorders, serum aldosterone levels are increased^31–37^. Thus, secondary hyperaldosteronism may contribute to or even account for the increased serum FGF23 levels in these disorders. Our data strongly suggest that increased FGF23 levels are a consequence of increased aldosterone levels in NCC KO mice. Vitamin D levels are similar in WT and KO mice, hence vitamin D seems not to be an important driver of FGF23 secretion in NCC KO mice. However, we find that PTH is increased in NCC KO mice, as previously reported^19^. Since PTH^38–40^ also stimulates FGF23 secretion, PTH may contribute to the increase of FGF23 in NCC KO mice.

The receptors for angiotensin II (AT1 and AT2)^41^ and aldosterone (MR)^42^ are readily expressed in the bone, which is the main site of FGF23 production. Surprisingly, these hormones, which have hitherto been implicated in regulating volume regulation in mammals, also have a function in bone homeostasis. Aldosterone is at least in part effective by stimulation of mineralocorticoid receptors with subsequent up-regulation of the serum- and glucocorticoid-inducible kinase 1 (SGK1)^20^, which activates NFκB, a transcription factor stimulating the expression of the Ca^2+^ channel Orai1^43^. Therefore, blocking of these receptors in NCC KO mice resulted in reversal of FGF23 phenotype. Wild type mice did not show significant reduction after blocking receptors indicating mineralocorticoid receptor blockers are effective reducing FGF23 levels only at pathological conditions (here higher aldosterone levels). NCC KO mice have activated renin-angiotensin-aldosterone axis^24,44^. At least in theory, it is possible that these hormones form a vicious cycle which may cause FGF23 upregulation in NCC KO mice. It will be interesting to analyze direct effects of renin/angiotensin on FGF23 levels in a separate study.

Several studies have previously shown NCC expression in osteoblasts or bone and suggested that inhibition of NCC activity may directly augment osteoblast differentiation^25,26^. In our study we have observed unchanged or even decreased transcript expression of FGF23 in primary osteoblasts following thiazide treatment. Therefore, our *in-vitro* data suggest that increased FGF23 levels in the serum of NCC KO mice are not merely a response of bone cells to direct NCC ablation but a consequence of lack of extra-osseous NCC activity.

To determine whether NCC KO mice adapt normally to a change from a normal-phosphate diet to a low-phosphate diet, animals were fed a low-phosphate diet for 5 days. There was, however, a marked alteration in hormone levels and mineral metabolism during the course of the low phosphate diet. Phosphate depletion led to a decline of serum phosphate concentration with enhanced calcium efflux from bone and increase of serum calcium concentration^45^. The hypercalcemia suppresses PTH release and thus abrogates the stimulatory effect of PTH on FGF23. The loss of PTH stimulation presumably accounts for the sharp decline of iFGF23 despite a further increase of serum aldosterone levels. Therefore, it is tempting to speculate that the stimulation by PTH may be a prerequisite for the effect of aldosterone on FGF23.

Our study indicates that NCC is not only an important player in sodium and volume homeostasis but also in the regulation of mineral metabolism. Thiazides have been shown to preserve mineralization in long and axial bones^46,47^. The mechanism appears to be multifactorial involving effects on the kidneys, intestinal calcium absorption^48,49^ as well as bone remodeling^25^. Previous findings in NCC KO^19,26^ mice are consistent with a randomized human trial showing hydrochlorothiazide treatment for 2 years decreased cortical bone loss in normal postmenopausal women^46^. Decreased urinary calcium output is a hallmark feature of thiazide treatment and could be explained by extracellular volume contraction with compensatory stimulation of NaCl and calcium reabsorption in proximal renal tubules and thick ascending limbs^50^. The current findings could also be of value in relation to mineral metabolism in human electrolyte disorders. Gitelman syndrome, a salt-wasting tubulopathy, is caused by mutations in the gene coding for the thiazide sensitive NCC^8,9^. The issue of renal phosphate wasting and hypophosphatemia has remained so far unrecognized with the exception of one controlled study and two case reports^51–54^. This is related to the fact that hypophosphatemia in these patients is very mild and physiological. Clearly, determinations of FGF23 and 1,25(OH)_2_D_3_ levels and a thorough investigation of renal phosphate handling in Gitelman’s patients is needed to determine if lack of NCC activity causes a similar phenotype in humans as we have observed in NCC KO mice.

## Methods

Unless specified otherwise, all chemicals and reagents were obtained from Sigma.

### Animal experiments

Animal experiments were conducted according to the Swiss law for the welfare of animals and were approved by the Veterinary authority of the Kanton Bern (Amt für Landwirtschaft des Kantons Bern Veterinärdienst). Experiments were performed in NCC KO and WT littermate mice. Generation, characteristics and genotyping of NCC KO have been described earlier^24^. Male age-matched mice (9–15 weeks old) were used for the experiments.

Mice had free access to control diet (Ssniff, Soest, Germany) containing 7000 mg/kg phosphorus or to phosphate-depleted diet (Altromin, Lage, Germany) containing 131 mg/kg phosphate and to tap water ad libitum. For blood draws, mice were anaesthetized with isoflurane (Roth, Karlsruhe, Germany) and blood was drawn into capillaries by puncturing the retrobulbar plexus. To measure urinary parameters, mice were placed individually in metabolic cages (Techniplast, Hohenpeissenberg, Germany) as described previously^55^. Mice were allowed a 2 days habituation period during which food and water intake, urinary flow rate and phosphate excretion were recorded every day to ascertain that mice were adapted to the new environment. Subsequently, consecutive 24 h urine collections were performed. Urine was collected under water-saturated oil. Urinary electrolytes and creatinine measurements were done at the Central laboratory of the Bern University Hospital.

Eplerenone was purchased from the Bern University hospital pharmacy (Eplerenone-Mepha, Switzerland). Mice on standard chow diet received 50 mg/kg of eplerenone through oral gavage at 8am and 8 pm for 7 days.

### Isolation and Culture of Primary Murine Osteoblasts

Primary osteoblasts were isolated from calvariae of one- and two-day-old C57Bl/6J mice by sequential collagenase digestion, as described previously^56^. Briefly, 5 calvariae were digested (5 × 20 min) in Hank’s Balanced Salt Solution (HBSS; Sigma, Buchs, CH) containing 3 mg/ml collagenase II (Worthington, NJ, USA). Fractions 3–5 were pooled, and 106 cells were seeded into 75 cm^2^ culture flasks and grown in culture medium containing αMEM (α-minimum essential medium) supplemented with 10% FBS, 100 U/ml penicillin, and 100 μg/ml streptomycin at 37 °C in a humidified atmosphere with 5% CO2. After 4 days in culture, cells were stored in liquid nitrogen (106 cells/ml). Before use, cells were thawed, grown in culture medium for 4 days, and used according to the experimental protocol; time periods for preparation and culture of primary calvarial osteoblasts were strictly adhered to.

### BBMV isolation

Renal cortical BBMV were prepared by the Mg^2+^ aggregation method as previously described^57^. Briefly, frozen kidney cortical samples were homogenized in ice-cold isolation buffer (300 mM Mannitol, 5 mM EGTA and 18 mM HEPES with pH adjusted to 7.5 with 1 M Tris base) containing fresh protease inhibitors, and crude membranes were obtained by centrifugation at 48,000 g for 1 h at 2 °C. Pellets were resuspended, homogenized in a Dounce glass homogenizer, and subjected to Mg^2+^ aggregation by addition of MgCl2 to a final concentration of 15 mM at 4 °C for 20 min. Aggregated membranes were removed by centrifugation at 3,000 g for 10 min at 2 °C and the supernatant was subjected to two additional rounds of Mg^2+^ aggregation as above, followed by centrifugation at 48,000 g for 30 min at 2 °C. The resulting pellet enriched in BBMV was dissolved in 200 µl ice-cold RIPA buffer. Total membrane fractions were prepared from frozen kidneys after homogenization in freshly made membrane preparation buffer (200 mM mannitol, 80 mM HEPES, 41 mM KOH, and 1 dissolved Complete Protease Inhibitor tablets, (Roche Diagnostics) using a homogenizer. The homogenate was then centrifuged for 15 min at 4 °C and 2,600 g, and the resulting supernatant was placed in an ultracentrifuge (Beckman ultracentrifuge tubes) for 1 h at 100,000 g and 4 °C. The pellet was resuspended in resuspension buffer, and protein concentration was quantified using a colorimetric assay (DcProtein Assay, Bio-Rad). 40 μg of dissolved BBMV protein or total kidney membrane protein were then used for separation by SDS-PAGE.

### Immunoblotting

Total protein (40 µg) was loaded on 8% v/v gels for protein separation by SDS-PAGE, followed by transfer to polyvinylidene difluoride (PVDF) membranes (Immobilon-P, Millipore Corporation, Bedford, MA, USA). Membranes were incubated overnight at 4 °C with primary antibodies. After washing, PVDF membranes were incubated with the appropriate secondary antibodies for one hour at room temperature. Immunoreactive bands were visualized using chemiluminescence (Amersham ECL, GE healthcare life sciences). Densitometric analysis was performed using ImageJ software (NIH, USA).

### Antibodies

The following primary antibodies were used: Polyclonal rabbit anti-NaPi-IIa 1:2,000, obtained from Prof. Biber J (University of Zurich)^58^. Polyclonal anti-actin (Santa Cruz Biotechnology, Santa Cruz, CA, 1:5000). FGFR1 mAb 9740: Cell Signaling (1:1000). Klotho MAb KM2076: Vendor Transgenics (1:2000). Secondary antibodies used were peroxidase-conjugated (Sigma-Aldrich, St. Louis, MO, USA; 1:20000).

### Total RNA isolation and cDNA synthesis

Total RNA was isolated from approximately 2 million cells using a Total RNA Mini Kit (Bio-Rad, Hercules, CA) according to the manufacturer’s protocol. First strand cDNA was synthesized from 2 μg of RNA using an iScript kit (Bio-Rad, Hercules, CA) according to the manufacturer’s instructions in 40 μl total volume.

### Quantification of mRNA expression

Quantitative real time PCR (qrtPCR) was performed with Applied Biosystems SYBR Green 2 × PCR Master Mix (Life Technologies, Carlsbad CA) in a System 7500 Fast thermal cycler using 1.5 μl of first strand DNA and 0.5 μl of 18 μM primer mixture in 10 μl total volume per well in duplicate. Reactions were performed in 384-well PCR plates and read on an ABI 7500 Fast instrument.

### Mouse Fgf23 primers used were

forward (5′-3′): TTTCCCAGGTTCGTCTAG

reverse (5′-3′): CTCGCAGGTGACTCT

### Mouse GAPDH primers used were

forward (5′-3′): TAACATCAAATGGGGTGA

reverse (5′-3′): GGTTCACACCCATCACAA

Calculated mRNA expression levels were normalized to the expression levels of GAPDH of the same cDNA sample. Relative quantification of gene expression was performed using the ΔCt method.

### Hormone measurements

Serum C-terminal-FGF23 and serum intact FGF23 (Immutopics International, San Clemente, CA, USA and Kainos laboratories, Japan respectively), serum 1,25(OH)_2_D_3_ (IDS, Boldon, UK), plasma intact PTH (1–84) (Immutopics, San Clemente, USA) and serum aldosterone (IBL, Hamburg, Germany) concentrations were measured by ELISA or EIA according to the manufacturer’s instructions.

### Statistics

Data are provided as means ± SEM, *n* represents the number of independent experiments. Statistical analysis was done using Student´s t test or one-way analysis of variance (ANOVA) with Tukey post-hoc test to correct for multiple comparisons, as appropriated. All statistical tests were two-sided and a P value < 0.05 was considered statistically significant.

## Electronic supplementary material

Supplementary file
