# Supplementary material for: Elevated FGF23 Levels in Mice Lacking the Thiazide-Sensitive NaCl cotransporter (NCC)
Source: Sci Rep. 2018 Feb 26;8:3590. doi: 10.1038/s41598-018-22041-1 (PMC5826922; doi:10.1038/s41598-018-22041-1)
Supplement: Supplementary file 1 — Supplementary file [file 41598_2018_22041_MOESM1_ESM.pdf]

Elevated FGF23 Levels in Mice Lacking the Thiazide-Sensitive NaCl cotransporter (NCC)

Ganesh Pathare[1, 2, 3*], Manuel Anderegg[1, 2, 3], Giuseppe Albano[1, 2, 3], Florian Lang[4,5], Daniel G. Fuster[1, 2, 3]

[1]Division of Nephrology and Hypertension, Bern University Hospital, University of Bern, Switzerland.

[2]Institute of Biochemistry and Molecular Medicine, University of Bern, Switzerland,

[3]Swiss National Centre of Competence in Research (NCCR) Transcure, University of Bern, Switzerland.

[4]Department of Cardiology, Vascular Medicine and Physiology, Eberhard-Karls-University of Tübingen, Germany

[5]Department of Molecular Medicine II, Medical Faculty, Heinrich Heine University, Düsseldorf, Germany

[*]Address correspondence to:
Ganesh Pathare
Institute of Biochemistry and Molecular Medicine,
Bühlstrasse 28, 3012, Bern, Switzerland
Email: ganesh.pathare@ibmm.unibe.ch
Phone: ++41 (0)31 632 31 44
Fax: ++41 (0)31 632 97 34

Supplemental Figures:

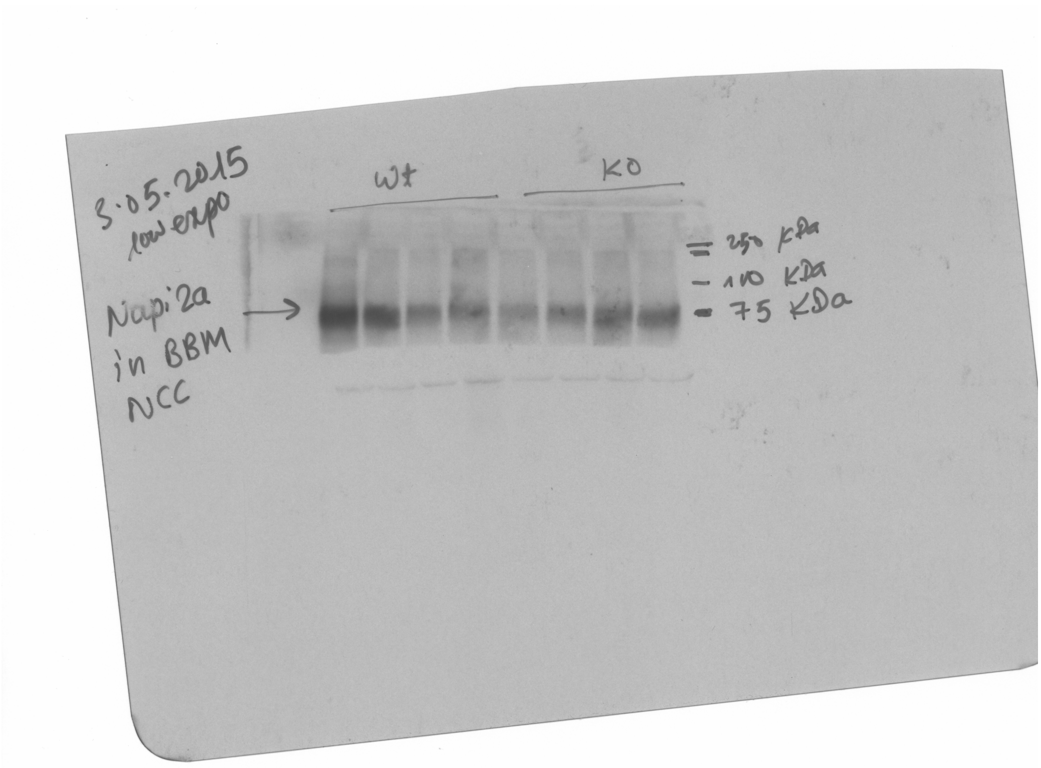

Fig. S1: Original uncropped western blot of Napi2a in BBM isolated from NCC WT and KO kidneys.

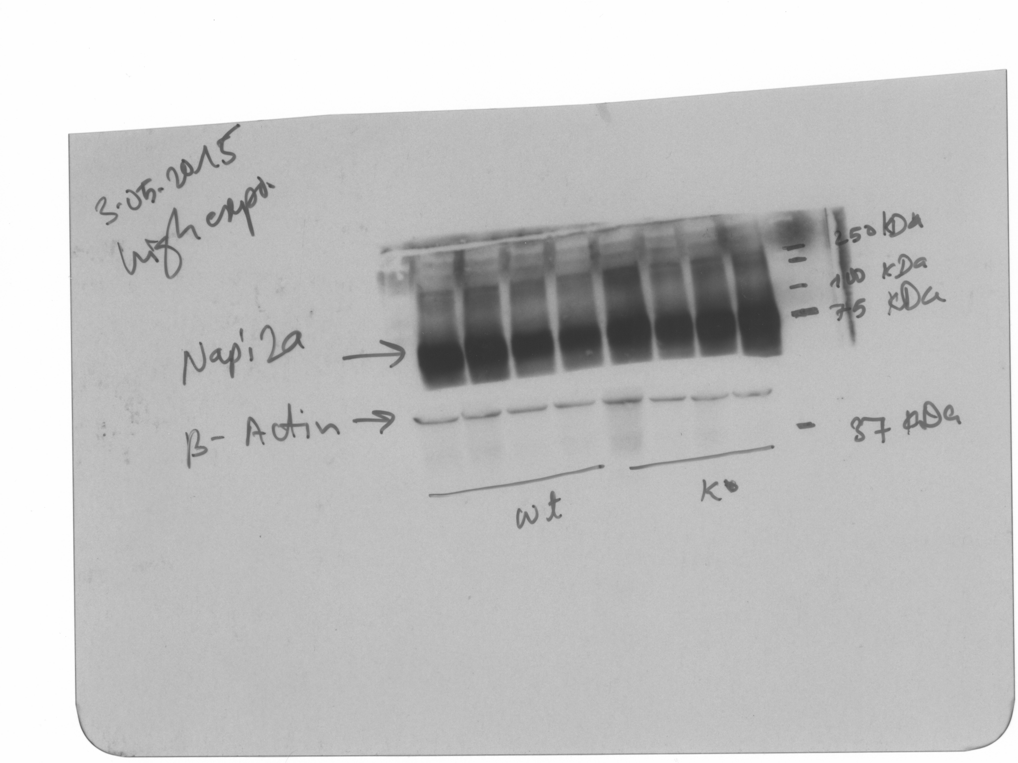

Fig. S2: Original uncropped western blot beta-actin in BBM isolated from NCC WT and KO kidneys.

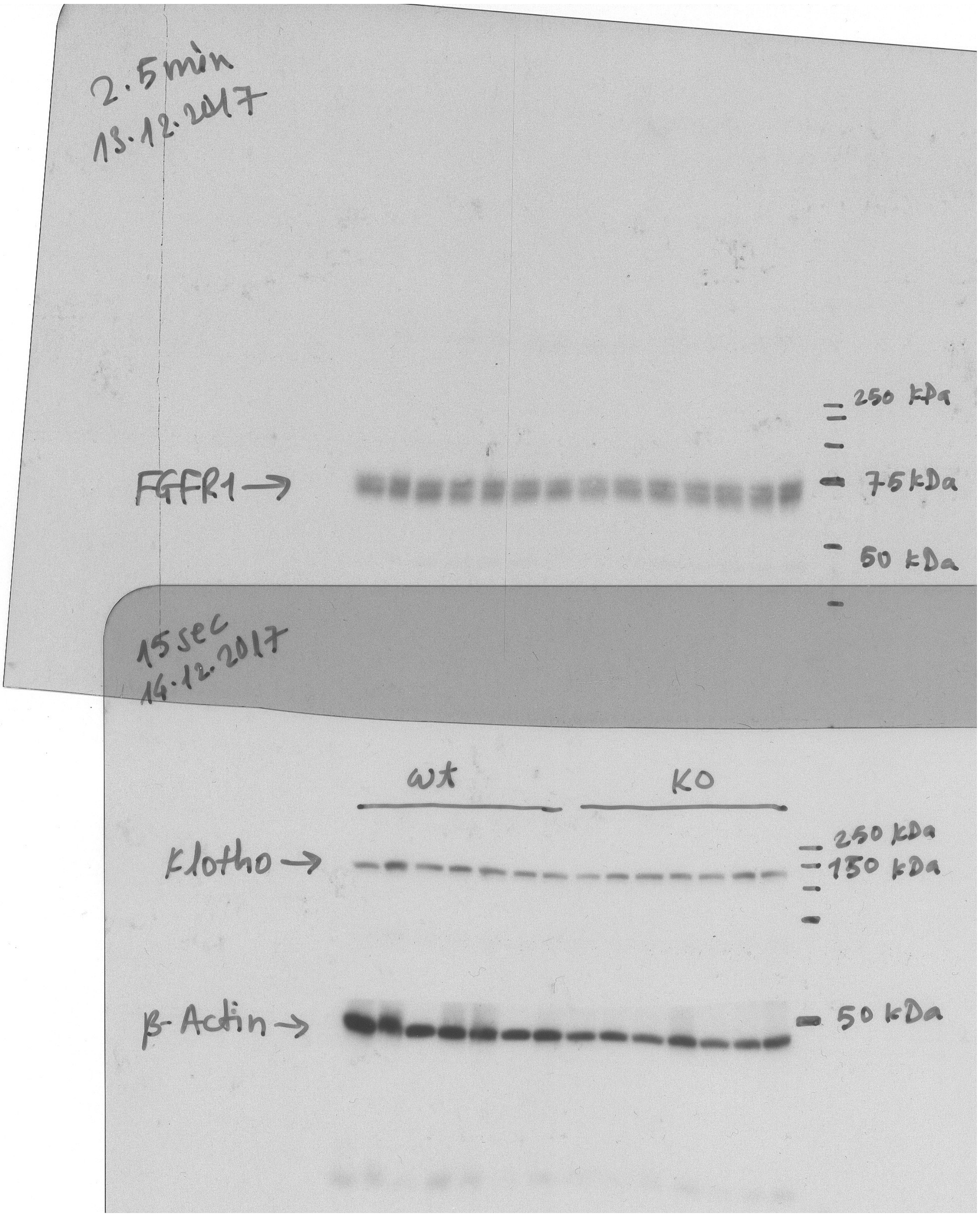

Fig. S3: Original uncropped western blot of FGFR1, Klotho and beta-actin from NCC WT and KO total kidney lysates.
